# Insights from TPPP3 and its family member proteins in neuronal diseases

**DOI:** 10.4103/NRR.NRR-D-25-00345

**Published:** 2025-08-13

**Authors:** Mishal Rao, Kun-Che Chang

**Affiliations:** 1 Department of Ophthalmology, Louis J. Fox Center for Vision Restoration, University of Pittsburgh School of Medicine, Pittsburgh, PA, USA; 2 Department of Neurobiology, Center of Neuroscience, University of Pittsburgh School of Medicine, Pittsburgh, PA, USA; 3 Department of Bioengineering, University of Pittsburgh, Pittsburgh, PA, USA

**Keywords:** axonal regeneration, glaucoma, microtubule stabilization, neuroprotection, Parkinson’s disease, TPPP3

## Abstract

Tubulin polymerization-promoting protein family member 3 (TPPP3) is a neuronal-specific protein involved in cytoskeletal stability, axonal maintenance, and neuronal survival. Dysregulation of TPPP3 is implicated in neurodegenerative diseases such as Parkinson’s disease and diabetic retinopathy. Unlike TPPP1, which is oligodendrocyte-specific, TPPP3 was reported to primarily promote neuronal regeneration and serve as a therapeutic target for neurodegenerative diseases such as Parkinson’s disease and glaucoma. Beyond the nervous system, TPPP3 has been linked to oncogenesis and tissue regeneration, suggesting potential roles in tumor suppression and wound healing. This review summarizes neuronal functions of TPPP3, therapeutic opportunities, and future research directions. Understanding the molecular mechanisms underlying function of TPPP3 could provide valuable insights into its therapeutic applications in neuroprotection.

## Introduction

Tubulin polymerization-promoting protein family member 3 (TPPP3) is a microtubule-associated protein that belongs to the TPPP family, which includes TPPP1 and TPPP2 (Vincze et al., 2006). Members of this family play a crucial role in the stability and assembly dynamics of microtubules, which are essential components of the cytoskeleton, and involved in neuronal function, axonal transport, and structural maintenance (Tokesi et al., 2010; Lasser et al., 2018). Dysregulation of microtubule-associated proteins can result in cytoskeletal destabilization, a hallmark of neurodegenerative diseases such as Parkinson’s disease (PD) and Alzheimer’s disease (Dubey et al., 2015).

TPPP3 or p20 is a microtubule-associated protein that regulates tubulin polymerization, cytoskeletal integrity, and neuronal homeostasis (Vincze et al., 2006). Unlike TPPP1 or p25, TPPP3 is primarily expressed in oligodendrocytes, influences mitotic spindle dynamics (Takahashi et al., 1993; Lehotzky et al., 2010), and contributes to remyelination (Kovacs et al., 2004). TPPP3 is selectively involved in neuronal cytoskeletal organization and axonal growth (Huang et al., 2017; Olah et al., 2022; Rao et al., 2024). Additionally, its expression in developing neural tissues highlights its fundamental role in neurogenesis and axonal maintenance (Rao et al., 2024; **Table 1**).

**Table 1:** Neuronal expression and functional characterization of the TPPP family member proteins

	Species	Expression	Neural Disease Link	Neural functional consequences	Potential therapeutic implications	References
TPPP1/219 AA/24 kDa	Human	Brain, neurons, oligodendrocytes	MSA, PD, DLBD, MS	Associates with SYN in Lewy bodies, glial, and neuronal inclusions; increased in normal area white matter/periplaque white matter oligodendrocytes but lost within demyelinated plaques in MS; essential for oligodendrocyte differentiation and myelination	Target for SYN modulation in PD/MSA; marker for oligodendroglial changes in MS; potential for remyelination in demyelinating disease	Kovacs et al., 2004; Baker et al., 2006; Hoftberger et al., 2010; Lehotzky et al., 2010
	Rat	Oligodendrocytes, brain, myelinating oligodendrocytes in development	Colocalizes with SYN in affected brain regions	Stabilizes microtubules, supports hippocampal fiber integrity, and regulates myelination	Relevant to oligodendrocyte function, neurodegeneration, and development	Takahashi et al., 1993; Kovacs et al., 2004; Skjoerringe et al., 2006
	Mouse	Oligodendrocytes, neurons	–	Likely contributes to microtubule stabilization and myelination	Potential role in neuroprotection	Kovacs et al., 2004
	*In vitro*	Expressed in mitotic cells, neurons, and oligodendrocytes	–	Promotes tubulin polymerization, inhibits mitotic spindle formation	Potential target in neurodegeneration and synucleinopathies	Tirian et al., 2003; Lehotzky et al., 2010; Schofield et al., 2013
TPPP2/ 170 AA/ 18.68 kDa	–	Not studied in neural tissue	Unknown	Unknown	Linked to sperm development and motility	Orosz, 2024
TPPP3/ 176 AA/ 18.98 kDa	Human	Retinal ganglion cells, brain	Associated with tubulin polymerization, tumor suppressor	Inhibits cell proliferation and migration	TPPP3 does not display an aggregation-promoting potency, potential therapeutic target for cancer treatment	Olah et al., 2022; Xu et al., 2023; Mo et al., 2024; Rao et al., 2024
	Mouse	Retinal ganglion cells	Potential role in optic nerve regeneration	Promotes axon regeneration	Possible therapeutic target for optic nerve repair	Rao et al., 2024
	Zebrafish	Brain, retinal ganglion cells	Regulated by microRNA-133b	Promotes axon regeneration	Possible therapeutic target for optic nerve repair	Huang et al., 2017

“–” indicates not reported, unknown, or not studied in the neural context. DLBD: Diffuse Lewy body disease; MS: multiple sclerosis; MSA: multiple system atrophy; PD: Parkinson’s disease; SYN: α-synuclein; TPPP: tubulin polymerization-promoting protein.

Emerging evidence highlights TPPP3 as a crucial regulator of neuronal survival and axonal repair, making it a promising therapeutic target for neurodegenerative diseases such as glaucoma and other optic neuropathies. By binding tubulin and promoting microtubule polymerization, TPPP3 ensures structural stability in neurons, a function particularly relevant for neuronal regeneration, where microtubule stabilization is necessary for axonal extension. Additionally, TPPP3 has been implicated in cell division and proliferation, with some studies suggesting context-dependent roles in oncogenesis. For instance, TPPP3 has been shown to suppress tumor progression in oral squamous cell carcinoma, while promoting growth and metastasis in lung and breast cancers (Zhou et al., 2010a; Ren et al., 2020; Xiao et al., 2025). However, this review focuses on its function during neuronal repair, underscoring its potential as a therapeutic target in neurodegeneration.

**Figure 1:**
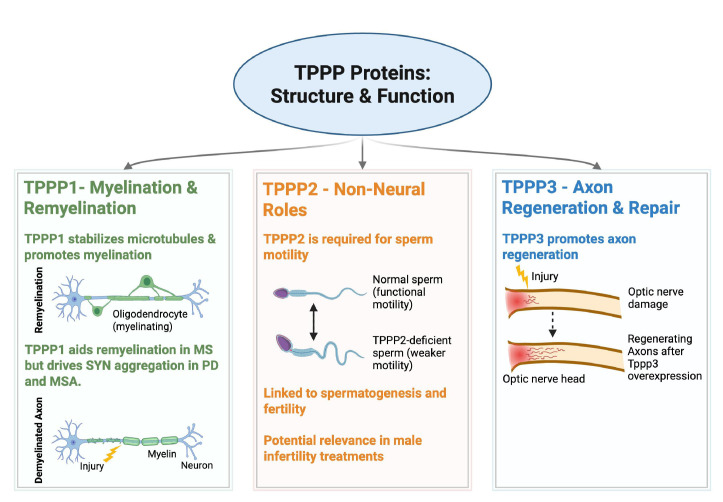
TPPP1 & TPPP3: linking myelination, axon growth, and neural repair. Created with BioRender.com. MS: Multiple sclerosis; MSA: multiple system atrophy; PD: Parkinson’s disease; SYN: α-synuclein; TPPP: tubulin polymerization-promoting protein.

This review will explore the role of TPPP3 in neuronal diseases, focusing on its interactions with microtubules, influence on neuronal differentiation, and neuroprotective mechanisms (**Figure 1**). Additionally, we will examine the potential of TPPP family member proteins as a therapeutic target for neurodegenerative diseases and CNS repair strategies.

## Search Strategy

In this narrative review, a literature search was conducted using PubMed and Google Scholar with the keywords: “TPPP1,” “TPPP2,” “TPPP3,” “microtubule stabilization,” “axon regeneration,” “oncogenesis,” “neurodegenerative diseases,” and “neurodegeneration.” Articles were first screened by their abstract, and those relevant to the scope of this review were evaluated in full. Studies were selected based on their relevance to the roles of TPPP3 and its family member proteins in neuronal function and disease. In total, 54 articles were included, with the majority published between 2018 and 2025.

## TPPP Family and Microtubule Dynamics

All TPPP family member proteins share a conserved p25α domain that enables tubulin binding and microtubule regulation, contributing to cytoskeletal organization and intracellular transport (Vincze et al., 2006). This structural domain underscores their evolutionary conservation and functional relevance across species. Sequence analyses show this domain is evolutionarily highly conserved across vertebrate species, including humans, mice, and zebrafish. While invertebrate homologs of TPPP1, such as Drosophila’s Ringmaker, retain core binding motifs with functional similarity despite lower overall identity and similarity (Mino et al., 2016; Shi et al., 2019; **Table 1**). TPPP1, TPPP2, and TPPP3 vary in molecular weight and tissue expression. While TPPP1 plays a pivotal role in oligodendrocyte differentiation and myelination, and TPPP2 is primarily restricted to spermatogenesis, TPPP3 is enriched in neurons and critical for axonal extension and repair (**Figure 1**). This structural and functional divergence reflects conservation and specialization within the TPPP family, highlighting their relevance within different disease contexts.

Tubulin, the fundamental subunit of microtubules, is crucial for maintaining neuronal structure and function. The TPPP family is central to tubulin polymerization and cytoskeletal stability. TPPP1, a 25 kDa protein predominantly expressed in oligodendrocytes, has contributed to axonal myelination and microtubule organization (Lehotzky et al., 2010). Initial identification of the TPPP-tubulin interaction came through its co-purification with tau protein kinases from bovine brain, highlighting its functional importance in promoting tubulin polymerization and stabilizing microtubule structures critical for neuronal transport and integrity (Ota et al., 2014). Additionally, phosphorylation by glycogen synthase kinase 3 and protein kinase C isoforms regulates TPPP1 proteins, further emphasizing their significance in cytoskeletal maintenance (Tirian et al., 2003; Otzen et al., 2005; **Table 1**).

Developmental studies reveal TPPP1 expression correlates with oligodendrocyte differentiation and is present throughout rat brain development, underscoring its importance in cellular maturation and myelination (Kovacs et al., 2004; Skjoerringe et al., 2006; Mino et al., 2016). Evolutionarily, homologous proteins such as Ringmaker in *Drosophila* highlight conserved functions in synaptic development and microtubule structuring across species, underscoring the fundamental importance of these proteins in neuronal development and their potential relevance to understanding human neurological disorders (Shi et al., 2019). TPPP2, an 18 kDa protein, is another member of this family, has not been studied extensively in neural contexts, and is primarily linked to spermatogenesis and sperm motility (Orosz, 2024).

Emerging evidence highlights the critical role of TPPP3, a 25 kDa protein, in microtubule dynamics across neuronal and non-neuronal contexts. In neuronal cells, its expression is essential for maintaining microtubule organization, which is crucial for axonal regeneration. For instance, elevated levels of miR-133b, which negatively regulates TPPP3, impair axonal regeneration in zebrafish by disrupting microtubule stability (Huang et al., 2017; **Table 1**). Similarly, reduced TPPP3 expression in alveolar type 2 cells results in impaired microtubule organization and defective cytoskeletal remodeling during cell differentiation (Zhang et al., 2022), further illustrating its broad functional importance (**Table 1**).

## Tppp3 and Neurodegenerative Diseases

### Parkinson’s disease

PD is characterized by progressive dopaminergic neurodegeneration in the substantia nigra and the formation of Lewy bodies, which are composed of aggregated α-synuclein (SYN). Due to its structural diversity and ability to adopt multiple conformations, SYN is called a chameleon protein, contributing to various cellular processes (Uversky, 2003).

SYN plays a role in maintaining the synaptic vesicle pool and dopamine metabolism. These processes are essential for efficient neurotransmission and neuronal plasticity (Bellucci et al., 2016; Mochizuki et al., 2018). Both SYN and microtubule instability are implicated in neurodegenerative diseases such as PD. Given its role in stabilizing microtubules, Tppp3 may influence PD pathogenesis by preserving cytoskeletal integrity in affected neurons.

A potential pathogenic link between Tppp3 and PD involves its interaction with SYN, which disrupts microtubule function and contributes to synaptic dysfunction and neuronal loss. SYN oligomers inhibit tubulin polymerization and their accumulation is associated with microtubule disruption (Prots et al., 2018). While TPPP1 has been shown to interact with SYN and promote its aggregation (Kovacs et al., 2004; Xie et al., 2021), the role of Tppp3 in this process remains unclear. The role of Tppp3 in PD could extend beyond microtubule stabilization. Emerging evidence suggests that Tppp3 may influence SYN, a hallmark of PD pathology (Olah et al., 2022). Unlike TPPP1, TPPP3 does not bind and promote SYN aggregation (Olah et al., 2022). TPPP3 inhibits the formation of TPPP1-SYN complex and suggests a potential modulatory role in TPPP1-SYN interactions. Disruptions in microtubule integrity contribute to the formation of toxic SYN aggregates, and stabilizing this network through Tppp3 modulation could mitigate disease progression. Further studies are needed to elucidate whether Tppp3 directly interacts with SYN or modulates its degradation pathways, such as the ubiquitin-proteasome system or autophagy.

### Glaucoma and optic neuropathies

Glaucoma is a leading cause of irreversible blindness, characterized by progressive retinal ganglion cell (RGC) degeneration and optic nerve damage (Quigley, 2011). In glaucoma, RGC loss is exacerbated by cytoskeletal instability and impaired axonal transport (Guo et al., 2020; Dias et al., 2022). Axonal transport deficits and cytoskeletal instability contribute to disease progression, as RGC axons rely on an intact microtubule network for intracellular transport and survival (McKerracher and Rosen, 2015; Dias et al., 2022).

In our recent study, we identified that Tppp3 is highly expressed within RGCs and is a promising molecular target for RGC neuroprotection and axon regeneration (Rao et al., 2024). Utilizing adeno-associated virus 2-mediated overexpression, we demonstrated significant improvements in RGC survival and enhanced axonal regeneration following optic nerve injury. While the exact molecular mechanisms underlying these effects remain to be explored, preliminary findings suggest a potential involvement of bone morphogenetic protein 4 signaling and inflammation-related cellular pathways implicated in neural repair processes. Further investigation into these mechanisms may offer valuable insights for therapeutic strategies in optic neuropathies.

Microtubule-stabilizing properties of Tppp3 may confer neuroprotection in glaucoma by promoting axonal survival and axon regeneration. Although the mechanism remains to be explored, the known role of Tppp3 in cytoskeletal dynamics makes Tppp3 a promising candidate for therapeutic strategies to enhance microtubule stability and prevent neurodegeneration. This is significant when considering disorders involving damaged neural pathways, such as glaucoma. Although the precise function of TPPP3 in the physiology of glaucoma is unknown, it is most likely involved in preserving the integrity of the microtubules in the optic nerve, which affects the vulnerability of RGCs to glaucomatous damage (Weinreb et al., 2014).

Furthermore, a recent genome-wide association study identified TPPP3 as a genetic risk locus closely related to diabetic retinopathy, highlighting its potential as a pathogenic factor and therapeutic target for this disease (Mo et al., 2024).

## Expanding the Therapeutic Potential of Tppp3

Given its role in microtubule stabilization and neuronal survival, Tppp3 presents a promising therapeutic target for neurodegenerative diseases and CNS repair. Gene therapy has emerged as one of the most promising approaches for harnessing the neuroprotective properties of candidate molecules. As utilized in our previous study, adeno-associated virus 2 has been successfully used in models of retinal and neurodegenerative diseases, suggesting that Tppp3 overexpression via adeno-associated virus 2 could enhance axonal regeneration (Rao et al., 2024). Furthermore, bulk RNA sequencing of the whole retina 2 days after optic nerve trauma revealed that Tppp3 overexpression upregulated Cldn1 and Cldn2 expression.

CLDN1 and CLDN2 are components of tight junctions in the blood–retina barrier, playing key roles in cellular processes such as proliferation, differentiation, and response to injury (Wolburg et al., 2001; Venugopal et al., 2019). A recent study suggests that CLDN1 modulates myelination-related proteins and regulates oligodendrocyte functions (Chen et al., 2021). In many neurodegenerative diseases, demyelination leads to axonal degeneration and functional impairments, highlighting the essential role of myelination in signal transduction in the central nervous system (CNS) (Stassart et al., 2018). While numerous axon regeneration-associated molecules promote axon branching and extension, they often lack proper myelination, adding to impaired signal transmission (Uyeda and Muramatsu, 2020; Franklin et al., 2021). Notably, combining microglia depletion with a GPR17 inhibitor enhanced the myelination of regenerating axons after optic nerve trauma (Wang et al., 2020). Given that TPPP1 has been implicated in myelination and remyelination, it would be valuable to investigate whether TPPP3 plays a similar role (Orosz et al., 2004). Since remyelination failure is a hallmark of chronic neurodegenerative diseases, understanding the function of TPPP3 could provide insights into potential therapeutic interventions.

Another potential strategy involves small molecule modulation, where identifying compounds that enhance or mimic Tppp3 activity could provide a pharmacological means to support neuronal survival and regeneration. While no specific small molecules targeting Tppp3 have been identified, ongoing research should be directed at exploring potential candidates within microtubule-stabilizing pathways. Investigating interaction partners of Tppp3 within the microtubule-stabilizing network, such as tau, microtubule-associated protein 2, and tubulin acetylation regulators, could reveal druggable targets that enhance its neuroprotective functions. Furthermore, stem cell–based therapies complement these approaches by modulating Tppp3 expression. Modulating Tppp3 expression in transplanted neural progenitor cells or induced pluripotent stem cells may improve neuronal differentiation and integration, potentially enhancing functional recovery in CNS injury models.

## Non-Neuronal Roles of Tppp3

While Tppp3 is primarily known for its role in neuronal function and neuroprotection, emerging evidence indicates it has broader biological functions beyond the nervous system. Recent studies have implicated Tppp3 in diverse cellular processes, including cell division, oncogenesis, and tissue regeneration (Staverosky et al., 2009; Zhou et al., 2010b; Harvey et al., 2019; Xu et al., 2023; Rao et al., 2024; Goto et al., 2025). Tppp3 exhibits context-dependent roles in cancer biology, demonstrating both tumor-promoting and tumor-suppressive activities depending on the cancer type. For instance, Tppp3 suppresses proliferation and migration in oral squamous cell carcinoma, potentially inhibiting tumor progression (Xiao et al., 2025), whereas in other cancer types, it enhances tumor growth, proliferation, and metastasis (Zhou et al., 2010b; Li et al., 2018; Shen et al., 2021; Xu et al., 2023). In glioblastoma, elevated Tppp3 expression correlates with poor patient survival outcomes (Pang et al., 2019; Ren et al., 2020) and may influence tumor progression through modulation of the immune microenvironment via immune cell infiltration (Yang et al., 2020; Liu et al., 2024).

Beyond oncology, Tppp3 appears to play a role in wound healing and tissue regeneration. Its expression is upregulated in regenerative tissues following injury, indicating its potential involvement in cellular repair mechanisms (Zhang et al., 2022). Additionally, developmental studies have reported the expression of Tppp3 in embryonic tissues, highlighting its possible roles in cytoskeletal organization critical for cellular differentiation and organogenesis (Staverosky et al., 2009).

Further investigations are needed to clarify the mechanistic contributions of Tppp3 in these non-neuronal contexts and to explore its potential as a therapeutic target beyond neurodegenerative diseases. Understanding how Tppp3 regulates cellular processes outside the nervous system could provide valuable insights into novel therapeutic strategies.

## Future Directions

Despite growing insights into the role of Tppp3 in neuroprotection, several critical questions remain unanswered. One key area requiring further investigation is how Tppp3 interacts with other microtubule-associated proteins, such as tau and microtubule-associated protein 2, and whether these interactions contribute to synergistic mechanisms enhancing its stabilizing properties. Additionally, exploring the involvement of Tppp3 in intracellular signaling pathways beyond cytoskeletal stabilization, including mitogen-activated protein kinase/extracellular signal-regulated kinase, phosphoinositide 3-kinase/protein kinase B, or bone morphogenetic protein 4 signaling pathways, could provide valuable insights into its broader role in neuronal survival and regeneration. Given the modulatory relationship between TPPP3 and TPPP1, future studies should explore whether TPPP3 indirectly affects TPPP1-SYN aggregation dynamics. It is also essential to examine the long-term consequences of Tppp3 modulation, specifically whether prolonged overexpression causes any oncogenic characteristics, and affects neuronal plasticity or metabolic homeostasis, to ensure its safety and efficacy for therapeutic applications.

Advancing research in these areas will be pivotal for translating Tppp3-targeted therapies into clinical applications, offering novel strategies for neuroprotection and CNS repair. Addressing these knowledge gaps will help determine full therapeutic potential of Tppp3 and contribute to a deeper understanding of microtubule-associated proteins in neurodegenerative disease mechanisms.

Further comparative studies on the TPPP family members will be essential to define their functional divergence and evolutionary conservation, which may uncover novel roles for Tppp3 in CNS pathology and repair.

## Conclusion

Tppp3 is emerging as a crucial regulator of microtubule dynamics, neuronal survival, and axonal regeneration. Its role in stabilizing cytoskeletal integrity positions it as a key player in neurodegenerative diseases such as PD and glaucoma, where disruptions in microtubule networks contribute to disease progression. Beyond its neuronal functions, Tppp3 has been implicated in oncogenesis, tissue regeneration, and developmental biology, suggesting broader biological significance. By advancing our understanding of Tppp3, we can pave the way for novel therapeutic strategies targeting microtubule stabilization and axonal repair in CNS disorders.

## Data Availability

*Not applicable*.
